# High Blood Pressure and Its Associated Factors Among Aksum University Students, Northern Ethiopia, 2019: A Cross-Sectional Study

**DOI:** 10.3389/ijph.2024.1607275

**Published:** 2024-05-20

**Authors:** Gebreamlak Gebremedhn Gebremeskel, Teklehaimanot Gereziher Haile, Gebremeskel Tukue Gebrewahd, Degena Bahrey Tadesse

**Affiliations:** ^1^ Department of Adult Health Nursing, School of Nursing, College of Health Sciences, Aksum University, Axum, Tigray, Ethiopia; ^2^ Department of Maternity and Neonatal Nursing, School of Nursing, College of Health Sciences, Aksum University, Axum, Tigray, Ethiopia; ^3^ Department of Critical Care Nursing, School of Nursing, College of Health Sciences, Aksum University, Axum, Tigray, Ethiopia

**Keywords:** associated factors, Ethiopia, high blood pressure, prevalence, hypertension

## Abstract

**Objectives:**

This study aimed to assess the burden of high blood pressure and its associated factors among students at Aksum University.

**Methods:**

A total of 240 participants were included; participants were selected through simple random sampling from May 2019 to July 2019. Logistic regression analysis was performed, with statistical significance set at a *p*-value <0.05 and a 95% confidence level.

**Results:**

This study found that 17.9% of the participants had high blood pressure, with higher rates observed in males (62.79%) than in females (37.21%). Several factors were identified as associated with high blood pressure, including a family history of high blood pressure [AOR 1.72, 95% CI (1. 75–4.04)], regular physical exercise [AOR 0.64, 95% CI (0.30–0.94)], alcohol consumption [AOR 2.16, 95% CI (1.07–4.62)], tobacco smoking [AOR 5.46, 95% CI (1.98–15.07)], and central obesity [AOR 2.72, 95% CI (1.12–6.58)].

**Conclusion:**

This study reveals that one out of six students had high blood pressure. Factors such as a family history of high blood pressure, physical inactivity, tobacco smoking, and central obesity were associated with this condition.

## Introduction

Hypertension (HTN) was defined as a systolic blood pressure of 140 mm Hg or higher and/or a diastolic pressure of 90 mm Hg or higher or current use of antihypertensive medications [1]. Cardiovascular diseases (CVDs), primarily caused by atherosclerosis (such as heart attack and stroke), are responsible for approximately 20% of deaths worldwide, with arterial hypertension being a major contributing factor. In developed countries, CVDs are the leading cause of death, accounting for 50% of all deaths. With nearly 16% of all deaths, they rank third and are becoming a significant public health concern in developing countries. Some countries, such as Singapore, Cuba, Argentina, Mauritius, Sri Lanka, and Uruguay, Chile, Trinidad and Tobago, have CVD as the leading cause of death [2].

Sub-Saharan African countries are currently undergoing rapid epidemiological transitions, characterized by increasing urbanization and lifestyle changes, which have led to an increase in noncommunicable diseases, particularly cardiovascular complications such as hypertension [3]. Although one study in Ethiopia indicated a decrease in deaths due to noncommunicable diseases, including hypertension [4], other systematic reviews have reported a high incidence of communicable diseases and associated risk factors. This double burden necessitates attention from policymakers and planners [5].

Hypertension is the most common cardiovascular disease and a significant public health problem in both developed and developing countries [6]. Hypertension, when combined with other risk factors such as khat chewing, age, increased body mass index (BMI), and smoking, increases the risk of developing cardiovascular complications. It is estimated that more than 95% of hypertensive patients have no identifiable cause (primary hypertension), while only a small percentage have an identifiable cause [2, 7]. Studies conducted in Kuwait among college students have shown a hypertension incidence of 7%, with higher rates among male students than female students [8]. A cross-sectional study in Saudi Arabia among university students revealed a hypertension prevalence of 7.5% out of 610 students [9]. Another cohort study conducted among university students at Al-Quds University in the West Bank reported a hypertension prevalence of 2.2% (3.3% among males and 0.4% among females), with obesity and smoking being associated factors [10]. In Brazil, a study among college students reported an elevated blood pressure prevalence of 9.7%, which was greater among males. Additionally, they found an 18.2% rate of excess weight, and an increase in body mass index (BMI) was associated with elevated mean blood pressure [11]. The prevalence of hypertension was 26.5% among students at Damietta University and 18.1% among students at Port-Said University [12]. Modifying risk factors associated with hypertension is crucial for preventing this condition and achieving better blood pressure control [13]. Currently, hypertension is a major health problem in developing countries. However, there is a lack of adequate studies in Ethiopia focusing on hypertension and its determinants among university students. Therefore, this study aimed to assess the prevalence of hypertension and its determinants among students at Aksum University in Ethiopia.

## Methods

### Study Area

The study was conducted at Aksum University, located in the Tigrai Regional State, approximately 1,024 km away from Addis Ababa, the capital city of Ethiopia. The university is situated in the western part of the town, specifically in the locality known as “Sefho.” University construction commenced in May 2006. Aksum University comprises four campuses, namely the main campus, referral hospital, Adwa campus, and Shire campus, and is home to 10 faculties and a total of 60 departments. According to the data obtained from the registrar’s office of Aksum University, a total of 10,086 regular students were enrolled in 2019. The university offers a wide range of undergraduate and postgraduate programs, including medicine, law, social sciences, languages, natural sciences, computational science, agriculture, engineering, health sciences, business, and economics.

### Study Design and Study Period

A cross-sectional study design was used for data collection from May 2019 to July 2019.

### Source Population and Study Population

The source population for this study comprised all students enrolled at Aksum University, totaling 10,086 individuals. The study population included all students located on the referral campus, main campus, and Shire campus of Aksum University who were present on the campus during the data collection period.

### Eligibility Criteria

All regular students of Aksum University were eligible to participate in this study, while postgraduate and extension students were excluded.

### Sampling Method

A simple random sampling method was utilized to collect the required information from the total population of 10,086 students. The sample size was allocated proportionally to each year, employing a simple proportional allocation method. Subsequently, a lottery was created based on the students’ identification numbers. A total of 240 study subjects were selected by chance, ensuring representation from each year of study.

### Sample Size Calculation

We used a single proportion formula for calculating our sample size, [n = Z (α/2)2 p (1-p)/d2] based on the following assumption:

P = the prevalence from a study conducted at Gonder University (7.7%) [14] with a marginal error (d) of 5% and a 95% confidence level, the sample size was calculated as follows:

Where n = the minimum sample size needed, P = the proportion of hypertension in the sample, D = margin of error for sampling, and Z a/2 = the standard normal value at 1.96.

Therefore,

$$n=\frac{{\left(1.96\right)}^{2}\left(0.077\right)\left(1-0.077\right)}{\left(0.05\right)2}=109.2$$

To account for potential nonresponses, we added a nonresponse rate of 10% to the calculated value; 109.2 + (109.2*10%) = 120.

Considering the design effect, which was accounted for by multiplying the sample size by 2 (120*2 = 240), our final sample size was 240.

### Data Collection Procedures

For data collection, we employed a structured questionnaire and conducted physical measurements of weight, height, waist circumference, hip circumference, BMI (calculated based on weight and height), and blood pressure. The questionnaire used was a modified version of the WHO Global Risk Factor Surveillance Questionnaire [15].

Weight measurements were taken using Seca weighing scales, adjusted to zero between each measurement. Participants were asked to stand without shoes, and their weight was recorded to the nearest 100 g. Participants wore light clothing and were not wearing shoes.

Height measurements were taken to the nearest 0.5 cm using a standard stadiometer. Participants stood upright, without shoes. To ensure proper positioning, participants were instructed to stand straight and look forward, and the data collector positioned the head so that the temporomandibular joint was level with the eyes and both heels were on the ground.

Blood pressure was measured twice while participants were in a sitting position. A standard mercury sphygmomanometer and an appropriate cuff size, covering two-thirds of the left upper arm, were used. Participants rested for at least 5 minutes before the study was conducted and refrained from smoking or consuming caffeine for 30 min prior. The second blood pressure measurement was taken 5 minutes after the first, and the average of the two measurements was used.

Waist circumference was measured using a nonelastic tape measure at the level of the iliac crest. Hip circumference was measured at the maximum circumference of the hip [16].

### Operational Definitions

✓ High blood pressure/hypertension: Average systolic blood pressure readings ≥140 mmHg and/or diastolic blood pressure readings ≥90 mmHg [1].
✓ Central Obesity: patients with a waist-to-hip ratio (WHR) greater than 1.0 for men or greater than 0.85 for women [15].
✓ Physical exercise: Regular intensity activities that require moderate physical effort and cause slight increases in breathing or heart rate for at least 150 min per week [15].

### Data Quality Control

The questionnaire was translated into the Amharic language and back-translated to English to ensure consistency. Four BSc nurses were recruited as data collectors, and they received training 1 week prior to the survey. Pretesting was conducted on 5% of the participants at the Soloda College Shire campus. At the end of each day, the collected data were checked for consistency, completeness, clarity, and accuracy by the principal investigator and supervisor.

### Data Processing and Analysis

Data coding was performed at the end of each day of data collection. The data were entered and cleaned using EPI info and then exported to SPSS version 22. Frequencies and random, independent checks were conducted to ensure the accuracy of the data entry. Univariate analysis was conducted using frequency distributions, percentages, tables, and charts to present the results. A binary logistic regression model was used, and all independent variables were tested with the dependent variable in bivariate analysis. Variables with a *p*-value of 0.25 or less in the bivariate analysis were included in the multivariable analysis to control for confounders. A *p*-value less than 0.05 indicated statistical significance at a 95% confidence level.

### Ethical Consideration

Our study method was conducted in accordance with relevant guidelines and regulations. In adherence to the Declaration of Helsinki guidelines, we obtained written informed consent from all study participants. This ensured their voluntary agreement to participate in the interview and a clear understanding of the study’s purpose, potential risks and benefits, confidentiality and privacy considerations, as well as their right to withdraw or decline answering certain questions.

## Results

### Sociodemographic Characteristics of the Participants

**FIGURE 1:**
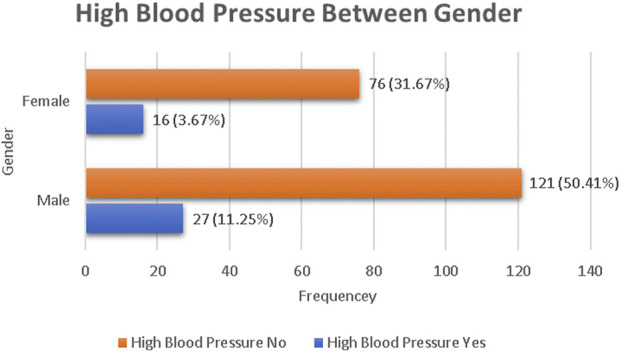
Frequency distribution of high blood pressure between gender in Aksum University students (Ethiopia, 2019).

A total of 240 respondents participated in this study, with a 100% response rate. Of the total respondents, 148 (61.7%) were males and 92 (38.3%) were females. The age of the participants ranged from 19 to 33 years. The 22–25 years-old age group constituted the largest group, with 121 (50.4%) study subjects. One hundred seventy-nine (74.6%) of the study subjects were Orthodox Christians. Of the respondents, 62.1% were from urban areas and 37.9% were from rural areas (Table 1). Forty-three (17.9%) of the respondents who took part in this study had elevated blood pressure. There was a greater prevalence of high blood pressure in male students than in female students, with an 11.25% prevalence (Figure 1).

**TABLE 1:** Descriptive characteristics of the study subjects in Aksum University students (Ethiopia, 2019).

Characteristics		No of subjects	% Of study subjects
Sex	Male Female	148	61.7
		92	38.3
Age in years	Mean ± SD	21.69 ± 1.83	
Ethnicity	Tigrai	110	45.8
	Amhara	67	27.9
	Oromo	30	12.5
	South	33	13.8
Religion	Orthodox	181	75.4
	Protestant	27	11.3
	Muslim	17	7.1
	Catholic	15	6.3
Marital status	Single	230	95.8
	Married	10	4.2
Monthly income	≤500	172	71.7
	500–1,000	46	19.2
	1,000–2000	22	9.2
Residence	Urban	149	62.1
	Rural	91	37.9

### Lifestyle-Related Factors and Medical History

52.7% of the respondents measured their blood pressure, while 114 (47.3%) of them had never measured their blood pressure previously. Among the total 240 respondents, 20% had a family history of hypertension. Among the study subjects, 10%, 8.3%, and 44.2% of the respondents reported chewing khat, ever smoking cigarettes during their lifetime, and consuming alcoholic drinks, respectively. A total of 14.2% of the respondents had central obesity (Table 2).

**TABLE 2:** Description of lifestyle related factors and medical history of study subjects in Aksum University students (Ethiopia, 2019).

Characteristics		Number of respondents	% Of respondents
Have you ever measured your blood pressure by health processional	Yes	126	52.7
	No	114	47.3
Have you ever have been told you have raised blood pressure	Yes	15	6.3
	No	225	93.8
Family history of HTN	Yes	48	20
	No	192	80
Have you ever been drinking an alcohol with in the past 12 months	Yes	106	44.2
	No	134	55.8
Have you ever been smoked cigarettes	Yes	20	8.3
	No	220	91.7
Khat chewing	Yes	24	10
	No	216	90
Regular physical exercise	Yes	128	53.3
	No	112	46.7
BMI	Mean ± SD	19.98 ± 2.28	
WHR	Mean ± SD	0.84 ± 0.07	
Central obesity	Yes	34	14.2
	No	206	85.8

Key: BMI, body mass index; HTN, hypertension; SD, standard deviation; WHR, waist to Hip Ratio.

### Factors Associated With High Blood Pressure

All the independent variables were computed first by binary logistic regression analysis, and those variables with a value less than 0.25 in the bivariate analysis were computed via multivariable analysis to control for confounders. According to the multivariable analysis, a family history of hypertension, regular physical activity, tobacco smoking, alcohol consumption, and central obesity were the variables significantly associated with high blood pressure (Table 3).

**TABLE 3:** Binary and multivariable analysis of the associations among Aksum University students (Ethiopia, 2019).

Characteristics		High blood pressure/HTN		COR		AOR	
		Yes	No	*p*-value	95% CI	*p*-value	95% CL
**Gender**	**M**	27	121	0.86	1.06 (0.54–2.11)	0.26	1.36 (0.63–3.20)
	**F**	16	76		1.0		1.0
**Age**				0.17	1.14 (0,95–1.36)	0.87	1.02 (0.82–1.27)
**Family history of HTN**	**Yes**	13	35	0.07	2.00 (0.95–4.23)	**0.01**	1.72 (1. 75–4.04) *
	**No**	30	162		1.0		1.0
**Alcohol drunk**	**Yes**	20	86	0.05	2.11 (1.0 5–4.24	**0.04**	2.16 (1.07–4.62) *
	**No**	23	111		1.0		1.0
**Tobacco smoking**	**Yes**	10	10	0.00	5.67 (2.19–14. 67)	**0.00**	5.46 (1.98–15.07) *
	**No**	33	187		1.0		1.0
**Chewing chat**	**Yes**	9	15	0.11	3.21 (1.30–7.93)	0.63	1.34 (0.39–4.52)
	**No**	34	182		1.0		1.0
**Regular physical exercise**	**Yes**	17	111	0.05	0.50 (0.26–0.99)	**0.03**	0.64 (0.30–0.94) *
	**No**	26	86		1.0		1.0
**Central obesity**	**Yes**	12	22	0.00	3.08 (1.839–6.86)	**0.02**	2.72 (1.12–6.58) *
	**No**	31	175		1.0		1.0

N.B: * indicates statistical significance, 1.0 for reference category, CI; confidence interval, AOR, adjusted odds ratio; COR, crud odds ratio; M, male, F, female; HTN, hypertension.

Bold *P*-values indicates they have a statistically significant association in the final model, multivariable analysis.

## Discussion

The prevalence of high blood pressure in our study population was 17.9%, which is higher than that reported in previous studies conducted among university students in Gonder (7.7%), Saudi Arabia (7.5%), and Eritrea (15.9%) [9, 14, 17]. However, our prevalence is lower than that reported in studies conducted at Damietta University (26.5%), Port-Said University (18.1%), and Qassim Saudi Arabia (29.2%) [12, 18]. These variations could be attributed to differences in study settings, sample sizes, lifestyle and behavioral characteristics of the participants, and the diagnostic criteria used for high blood pressure. In our study, we used the current WHO cutoff level of 140/90 mmHg, while some studies with lower prevalence used the former level of ≥160/90 mmHg.

More than half of the respondents (53.3%) reported engaging in regular physical exercise. Individuals who participated in regular physical exercise were 0.64 times less likely to develop high blood pressure than were those who did not exercise regularly. This can be explained by the increasing adoption of sedentary lifestyles and the use of motorized transportation in urban areas. Furthermore, as our study focused on students, prolonged sitting during study or teaching and learning processes may contribute to a higher risk of hypertension among sedentary individuals [19, 20].

Respondents with a positive family history of hypertension were 1.72 times more likely to have high blood pressure than those without a family history. These findings align with studies conducted in Sri Lanka and Japan [21, 22]. Additionally, our study revealed a significant association between alcohol consumption and high blood pressure, with alcoholic consumers being 2.16 times more likely to develop high blood pressure than non-consumers. This association may be explained by the detrimental effects of alcohol on lipid levels in the bloodstream, which can lead to arterial damage and increased blood pressure.

The presence of central obesity among university students was found to be associated with a 2.72-fold greater risk of developing high blood pressure. These findings are consistent with those of previous studies, indicating that central obesity significantly increases the likelihood of elevated blood pressure in this population. Similarly, tobacco use was also significantly associated with a 5.46-fold greater risk of developing high blood pressure among university students. These findings align with previous research findings, highlighting the strong association between tobacco use and elevated blood pressure [23, 24].

Lastly, although it is not statistically significant, we would like to provide additional remarks regarding the 10% of the study population, which corresponds 1 out of 10 students, reported khat chewing. This finding suggests that khat chewing is prevalent in these population. Considering its prevalence, further investigation is needed on the khat use pattern, including frequency, duration, and potential social and health implications. Additionally, education and awareness programs targeted at students should be considered to address the potential health risks associated with khat use and promote healthier lifestyle choices.

### Limitations of the Study

Although this study provides a valuable insight into high blood pressure and associated factors among Aksum University students, the nature of the cross-sectional design makes it difficult to establish causal relationships between high blood pressure and associated factors. Besides, the generalizability of the findings may be limited due to the specific population studied. Self-reported measures, such as lifestyle behaviors and medical history, may introduce potential information bias and confounding biases into the study findings. Lastly, we relied solely on anthropometric measurements and blood pressure readings without utilizing biochemical tests due to cost and availability constraints.

### Conclusion

Our study revealed a relatively high prevalence of high blood pressure among Aksum University students in northern Ethiopia. The identified factors associated with high blood pressure in this population included family history, inadequate regular physical activity, alcohol consumption, khat chewing, and central obesity.

### Recommendation

We recommend implementing health education programs that target modifiable risk factors such as alcohol consumption and promote regular physical exercise among university students. Additionally, screening initiatives should be undertaken to identify high-risk students for early intervention and prevention. Screening campaigns, such as those on hypertension days, could be organized, and high blood pressure students should be connected to appropriate healthcare facilities. Furthermore, further interventional research in this area should be conducted.

## Data Availability

The datasets used and analyzed during the current study are available from the corresponding author upon reasonable request.

## Abbreviations

CVD, Cardiovascular Disease; HTN, Hypertension; WHO, World Health Organization.
